# Dural Immune Cells, CGRP, and Migraine

**DOI:** 10.3389/fneur.2022.874193

**Published:** 2022-03-31

**Authors:** Louis K. Balcziak, Andrew F. Russo

**Affiliations:** ^1^Department of Molecular Physiology and Biophysics, University of Iowa, Iowa City, IA, United States; ^2^Neuroscience Graduate Program, University of Iowa, Iowa City, IA, United States; ^3^Department of Neurology, University of Iowa, Iowa City, IA, United States; ^4^Center for the Prevention and Treatment of Visual Loss, Veterans Administration Health Center, Iowa City, IA, United States

**Keywords:** meninges, immune, dura mater, migraine, CGRP, macrophage, perivascular

## Abstract

Migraine is the most common neurological disorder in the world, affecting 12% of the population. Migraine involves the central nervous system, trigeminal nerves and meninges. Recent advances have shown that targeting calcitonin gene-related peptide (CGRP) through either antibodies or small molecule receptor antagonists is effective at reducing episodic and chronic migraine episodes, but these therapeutics are not effective in all patients. This suggests that migraine does not have a singular molecular cause but is likely due to dysregulated physiology of multiple mechanisms. An often-overlooked part of migraine is the potential involvement of the immune system. Clinical studies have shown that migraine patients may have dysregulation in their immune system, with abnormal plasma cytokine levels either during the attack or at baseline. In addition, those who are immunocompromised appear to be at a higher risk of migraine-like disorders. A recent study showed that migraine caused changes to transcription of immune genes in the blood, even following treatment with sumatriptan. The dura mater is densely packed with macrophages, mast and dendritic cells, and they have been found to associate with meningeal blood vessels and trigeminal afferent endings. Recent work in mice shows activation and morphological changes of these cells in rodents following the migraine trigger cortical spreading depression. Importantly, each of these immune cell types can respond directly to CGRP. Since immune cells make up a large portion of the dura, have functional responses to CGRP, and interact with trigeminal afferents, CGRP actions on the dural immune system are likely to play key roles in migraine.

## Introduction

Migraine is a highly common neurological disorder characterized by intensive headaches. While almost everyone has headaches from time to time, what separates migraine is the duration, intensity and symptoms associated with the headache. It has been estimated that roughly 12% of the global population experiences migraine annually (1, 2). The International Headache Society (IHS) defines migraine as a recurring headache that lasts between 4 and 72 h. The headache occurs on one side of the head, and is accompanied by “pulsating quality, moderate or severe pain intensity, and aggravation by or causing avoidance of routine physical activity" (3). Additional diagnostic symptoms occur during the migraine attack, including vomiting, nausea, photophobia and phonophobia. Migraine patients experiencing an attack have a higher sensitivity to light (photophobia) and sound (phonophobia) that can cause discomfort, and this is one quality of migraine that makes it much worse than normal headache. Another important fact is that migraine is 2 to 3 times more common in women than men, suggesting a sex difference impacting the neurological environment (4). Approximately 20–30% of migraine patients also experience a disconcerting sensory phenomena called an aura prior to the headache (5).

Migraine is a multifaceted disorder involving the peripheral and central nervous systems (CNS) and the meninges, particularly the dura mater (6). There are many potential triggers of migraine, so migraine shouldn't be seen as having a singular cause but rather, migraine represents a spectrum of triggers, symptoms, and potential treatments (7). Within this complexity, it is generally accepted that trigeminal neurons are responsible for the pain of migraine. These neurons sense modalities such as pain, touch, temperature and mechanoreception (8, 9). Trigeminal sensory neurons innervate the face, jaw, and anterior portion of the head and dura mater (9). The nerve endings also innervate the sutures of the skull and can even be found to traverse the sutures into extracranial tissue (10, 11). The interface of nerve endings with both extracerebral and extracranial targets add to complexity studying the TG.

One trigger associated with migraine with aura is cortical spreading depression (CSD) (12). CSD causes an increase in trigeminal ganglion (TG) firing following application in animal models (13). A recent study found that migraine with aura patients have increased uptake of a radiolabeled inflammation-associated protein into the meninges and occipital skull bone (14). This provides strong evidence that CSD-induced meningeal inflammation in rodent models also occurs in migraine patients. The role of CSD and meningeal immune cells will be further discussed below.

The afferent endings of the TG interface with the dura mater, and the complex microenvironment composed of nerve endings, blood vessels, fibroblasts, and immune cells (15, 16). Early hypotheses of migraine were that migraine was caused by a vascular component (17, 18). Proponents of such theories date back to the classical Greek physician Galen. In the 1950's, Wolff postulated that migraine was due to distention of cranial vessels and vasodilation. He found that treatment with ergotamine compounds reduced the pulsing and partially restored function. Ergotamine is a serotonergic vasoconstrictor that also inhibits trigeminal nerve transmission (19). This helped lead to development of more specific acute treatments. One of the main acute treatments of migraine, sumatriptan (a 5HT1_B/D_ agonist) alleviates migraine pain and causes vasoconstriction (20). Sumatriptan also reduces synaptic release of neurotransmitters of trigeminal neurons (21). A counter to the argument that migraine is a vascular disorder comes from the development of non-vasoactive serotonergic medication such as lasmiditan. Lasmiditan is a 5HT_F_ agonist with no vasoconstriction side effect, at least *in vitro* (22). The efficacy of lasmiditan with no significant vascular effects suggests that blood vessels are only partially involved. An additional argument was based on the observation that while some vasodilators like nitroglycerin induce headache in migraine patients, another dilator, vasoactive intestinal peptide failed (23, 24). However, this argument has lost weight since a recent study with prolonged infusion of vasoactive intestinal peptide showed that it can induce migraine in patients (25).

Recent advances in the field have involved the neuropeptide CGRP. It is a 37 amino acid peptide released from trigeminal ganglion neurons that can induce vasodilation, nociception, and neurogenic inflammation (26–28). CGRP is released both in the periphery and centrally (26, 29, 30). Researchers noted that CGRP can induce migraine-like headache in migraine patients following infusion, including symptoms such as photophobia (31). Jugular vein CGRP is higher during migraine onset than in control subjects (32). Chronic migraine patients also have elevated levels of plasma CGRP at baseline compared to healthy controls (33). Using the preclinical and clinical evidence, scientists developed treatments targeting either CGRP or its receptor complex. The FDA has approved both CGRP-blocking or receptor blocking antibodies, as well as small molecule antagonists against the CGRP receptor (34, 35). Monoclonal antibodies result in a 50% reduction in migraine days in 50% of episodic migraine patients (36). The antibodies can also be partially effective in drug resistant migraine by reducing migraine headache days (4.2 fewer headache days in a month compared to baseline) (37). CGRP antibodies have fewer adverse side effects compared to other prophylactics (38).

Despite the success of recent CGRP-targeting medications, CGRP is not the only component of migraine induction and pathogenesis. The failure to ablate migraine, and only provide a reduction in migraine headache days suggests that CGRP is merely one player in the complex physiology of migraine. The physiology of the meninges should be taken into consideration, due to the complexity of migraine and headache disorders. This review will focus on clinical evidence of immune dysfunction in migraine patients, the anatomical and physiological relationships of the immune cells in the dura mater and their potential regulation by CGRP, and pre-clinical evidence implicating the immune system in migraine-like symptoms.

## Clinical Evidence of the Immune System Dysregulation in Migraine

Over the years evidence has emerged that migraine patients may have immune system dysfunction. Peripheral cytokine levels have been used to show a shift in the general inflammatory state of the body. One study showed that migraine patients have higher levels of interleukin 1-beta (IL1-β) and interleukin-6 (IL6), and lower levels of interleukin-10 (IL10) compared to healthy control patients (39). A 2015 study noted that migraine patients had higher IL6 levels compared to healthy controls (40). Tumor necrosis factor α (TNF-α) was elevated during attack in migraine patients with aura, and baseline levels were increased in general migraine patients. A 2021 study measured cytokine levels in the blood in migraine patients and healthy controls and discovered that TNF-α was elevated, but IL1-β was not compared to controls (41). Cerebrospinal fluid (CSF) protein measurements indicate that migraine patients have significantly different levels of transforming growth factor beta (TGF-β) 1, interleukin-1 receptor antagonist and monocyte chemoattractant protein 1 compared to controls (42). It is important to note that this is within the confines of the CNS, vs. peripheral blood levels that the previous studies have represented. Finally, while cytokine dysfunction in migraine is indicated by numerous studies, there are conflicting reports (39, 41).

Serum CGRP has been noted as either a potential biomarker or a poor marker due to variability in concentrations, patient conditions and diagnostic criterion. For example, Lee et al. found that healthy controls had a mean serum level of 75.7 picograms/mL CGRP, while episodic migraine patients showed 67.0 picograms/mL. In that study, chronic migraine patients had no increase in interictal levels of CGRP (43). But another study found that interictal CGRP levels were significantly elevated in chronic migraine patients (74.9 picograms/mL) compared to 46.37 picograms/mL in healthy patients (33). This discrepancy is highlighted in a Lancet review from 2021 (44). The lack of reproducibility could be explained in part due to different exclusion criteria, different ELISA sources, time of extraction, or methodology. The study by Cernuda-Mellon et al. and the one by Lee et al. did use similar timeframes, collection conditions, and measurement techniques (33, 43). More studies should be done to determine if CGRP is a valid biomarker of migraine.

Other evidence that doesn't rely on peripheral markers are comorbidities with immune disorders and migraine. Meta-analysis noted that headache had overlap with several autoimmune diseases like multiple sclerosis, rheumatoid arthritis, vasculitis, and allergic diseases (45). The authors hypothesize that headache or migraine phenotypes may be a “consequence of general inflammatory mechanisms involving meningeal vessels and activating trigeminal terminals, especially in individuals with a previous history of headache…” (45). A study investigating irritable bowel disorder (IBS) gave some evidence of a causal relationship between IBS and migraine (46). The potential dysfunction in cytokines in migraine patients, and abnormal CGRP activity may influence the gut-brain axis (47). It is important to note that CGRP doesn't just signal in the meninges, but all over the body, including the gastrointestinal (GI) tract. CGRP is postulated to influence the gut microbiome through interaction with resident GI tract immune cells as well as on gastroenteric motility (48, 49). This in turn may explain the correlation with migraine/headache in IBS disorders (47). There is also a significant association between celiac disease, an immune disease of the GI tract, and migraine (50).

Genome-wide association studies and single nucleotide polymorphism (SNP) studies provide additional evidence of the involvement of immune dysregulation in migraine. In a Jordanian population of 198 migraine patients and 200 controls, there was a significant association with two SNPs regarding TNF-α gene (51). There was also a decrease in circulating lymphocytes in the blood. Lymphocytes include cell types such as T and B cells (52). This is supported by a 2021 study showing that there is a significant decrease in peripheral regulatory T cells in migraine patients compared to controls (53). Changes to the immune system through different genes and SNPs may influence the environment of the dura mater, in parallel with the trigeminal afferent endings. In migraine patients, an RNA sequencing study in 2021 examined blood RNA levels during migraine attack or during the baseline period. Genes involved with the immune system, along with fat metabolism and signaling pathways were implicated in the study (54).

Measurement of peripheral gene expression through qPCR hints at immune dysfunction in migraine as well. A group of researchers examined migraine patients with or without aura compared to healthy controls and extracted jugular venous blood to measure gene expression of various cytokines (41). Migraine patients had elevated levels of interleukin-4 (IL4), TGF-β, TNF-α and interferon gamma. Some cytokines that were previously found to have higher circulating protein levels such as IL1-β were not significantly different from controls.

The evidence for immune system involvement is diverse, but not concrete enough to pinpoint a distinct role of these cells. But there does appear to be an involvement of this system, at least for part of the pathogenesis of migraine. The following section will highlight the immune cells present in the meninges, their actions, relation to trigeminal afferents and influence on neurons and blood vessels.

## Dura Mater Relevance and Overview

**Figure 1:**
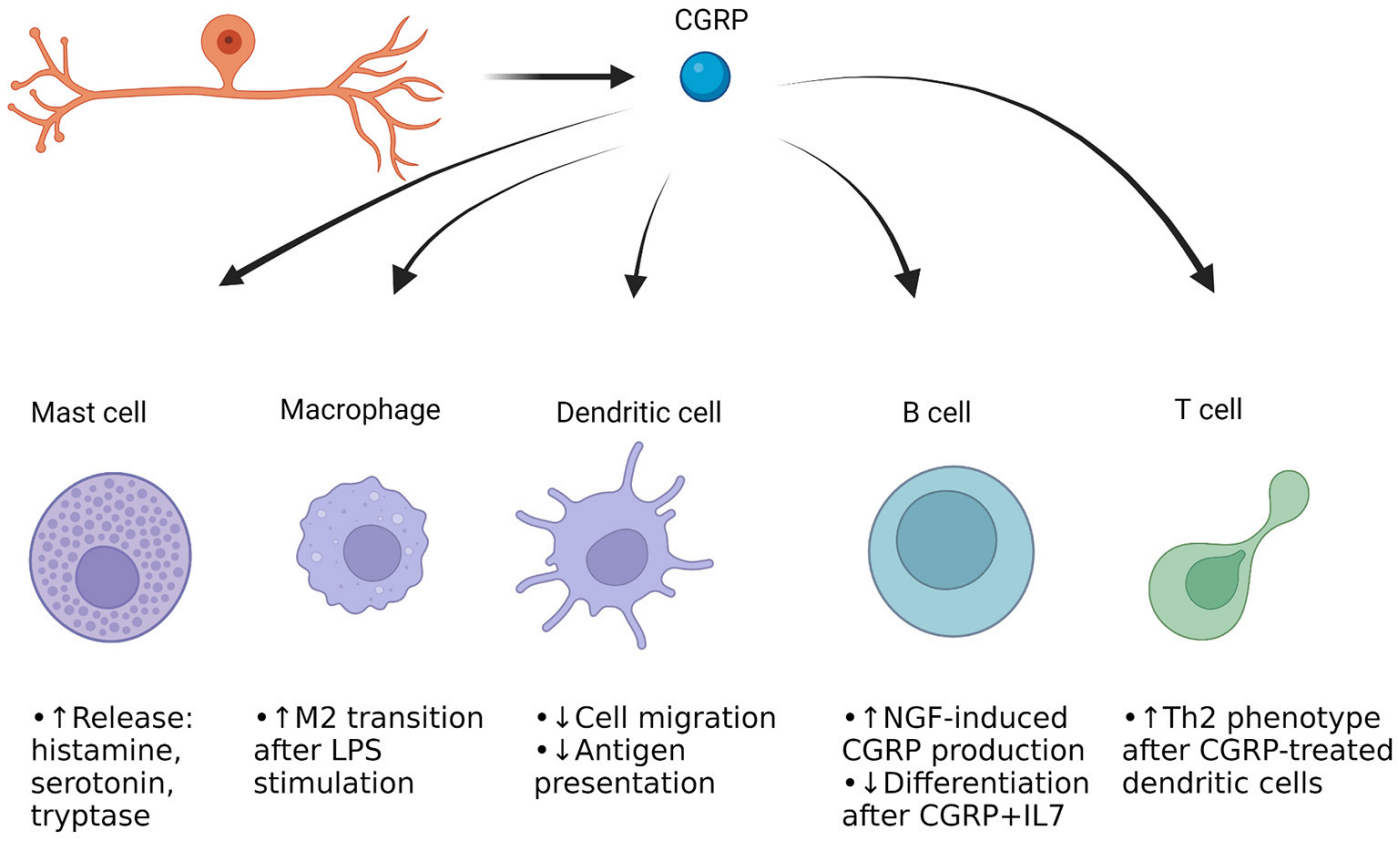
CGRP actions on immune cells in the dura mater of the meninges. Cells of both the innate and adaptive immune systems are present. Mast cells and macrophages represent the immediate frontline defense against pathogens and harmful compounds, while dendritic cells help move parts of the harmful pathogen/protein to the adaptive immune cells (71–73). The B cells can produce antibodies against the antigens, and the T cells can differentiate to either kill cells or aid fellow B and T cells for enhanced immune response. Most of the work of CGRP's effect on these cells has been done in culture, but it appears that CGRP induces neurogenic inflammation through mast cell degranulation, while enhancing anti-inflammatory macrophage function when stimulated with an inflammatory compound (27, 65, 66, 74). CGRP appears to reduce dendritic cell presentation of antigens (75). CGRP's effect in B cells and T cells is not well-studied but may drive the adaptive immune system to undergo physiological changes upon presence of certain triggers.

The dura mater is the outermost layer of the meninges that contains extracerebral blood vessels, fibroblasts, trigeminal afferent endings, sympathetic and parasympathetic efferent endings, and numerous immune cells (55–60). The trigeminal afferents extensively innervate the blood vessels and other regions of the dura mater, and interface with the extracellular matrix (ECM) (61). The interplay between afferent endings, vessels, immune cells, and ECM is hypothesized to be one way in which migraine is induced, particularly by neuropeptides like CGRP as well as other small molecules. This is referred as the trigeminovascular hypothesis of migraine (62). It is thought that there is initial trigeminal activation, resulting in release of neuropeptides like CGRP and Substance P into the trigeminovascular space. The trigeminal afferents also release small molecules such as glutamate (63). CGRP directly binds to vascular smooth muscle cells and causes hyperpolarization through metabotropic signaling and downstream phosphorylation of K_ATP_ channels by protein kinase A (64). This results in relaxation and thus vasodilation. CGRP also acts on resident immune cells such as macrophages and mast cells. The macrophages may become activated, and the mast cells degranulate (65, 66). This degranulation involves release of inflammatory chemicals such as histamine, proteases and various cytokines that are implicated in headache (67). Many of the compounds released can sensitize trigeminal afferents, leading to an increase in firing (68). Macrophage activation may result in release of cytokines that could further sensitize afferents (69). It should be noted that CGRP itself can sensitize afferent endings to compounds such as ATP, so there could be a synergism with cytokines and CGRP regarding afferent sensitization (70). The following section will discuss each of the immune cells present in the dura mater, interactions with trigeminal afferents and CGRP, and how they could be involved in migraine. Figure 1 provides a general overview of the immune cells present in the meninges as well as CGRP's actions on these cells.

## Immune Cells in the Meninges

### Macrophages

Macrophages are monocytic lineage cells that act as frontline defenders through the innate immune system (71). In the dura mater, the macrophages are derived from bone marrow and replenish over time (76). They are highly mobile and engage in phagocytosis of pathogens or dying cells (77). Other functions of macrophages include tissue repair and remodeling and cytokine release (78). A 2017 study by McIlvried et al. found that in the rodent dura mater, over 17% of cells present are immune cells, and of these almost two-thirds are macrophages (79). The high proportion of cells in the dura being immune cells (roughly 1 in 10 dural cells being a macrophage) represents a dynamic and ever-changing portion of the dura. Research has shown that mouse macrophages interact with cultured trigeminal ganglion cells in either a genetic model or wild type mice (80). They found that macrophages underwent more phagocytosis of particles in co-culture with TGs of either genotype compared to no co-culture. This suggests that macrophages may have a physiological relationship with TG neurons.

**Figure 2:**
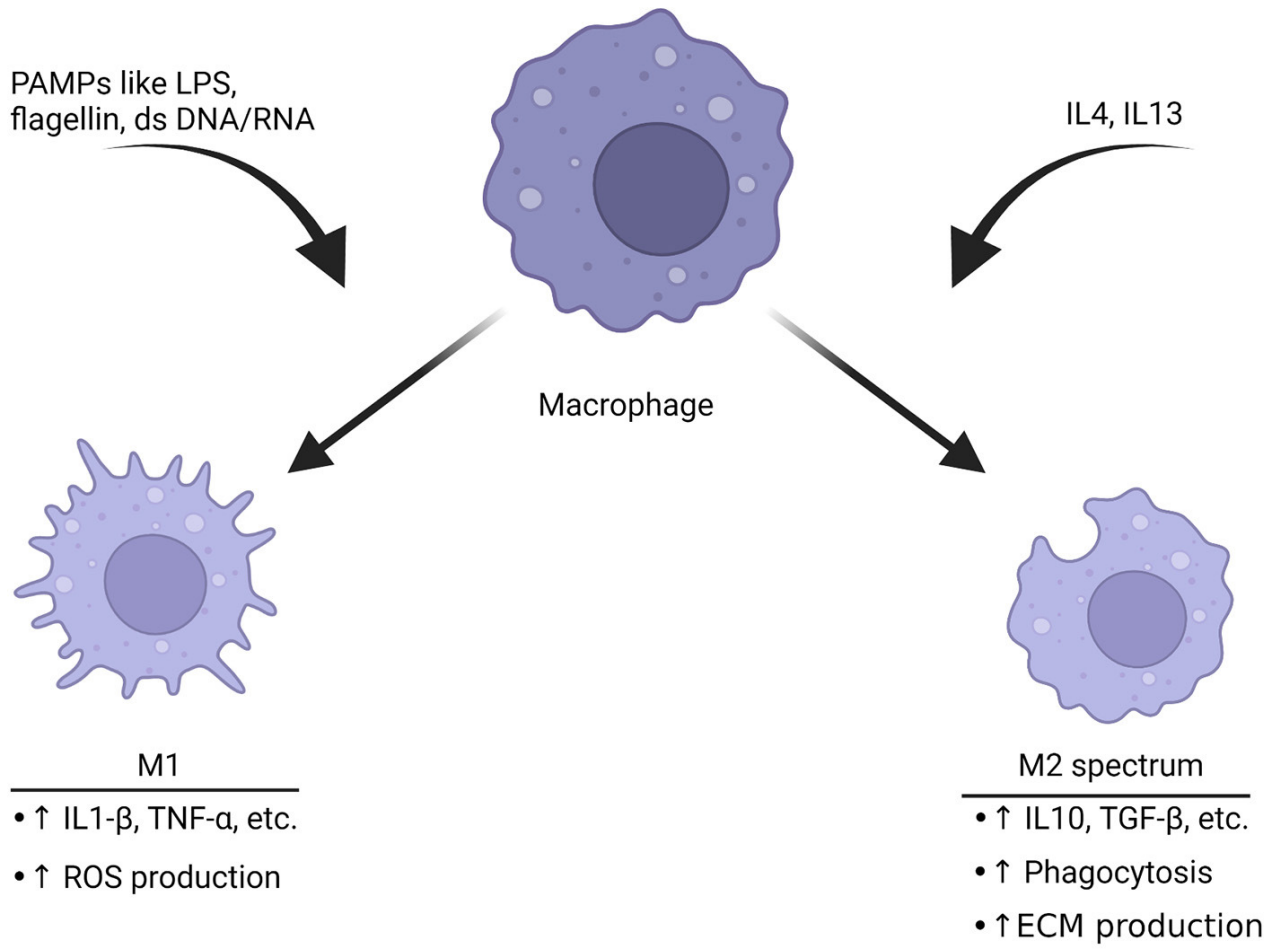
Macrophages polarize toward different phenotypes depending on the stimuli. Triggers like pathogen-associated molecular patterns (PAMPs) including LPS, double-stranded DNA or RNA can induce M1 polarization. This phenotype is characterized by production of inflammatory cytokines and an increase in ROS production to help break down targets. The M2 spectrum of polarization is activated by cytokines such as IL4 or IL13. The result is a transformation into the M2 spectrum (M2a, b, c, and d) which is characterized by enhanced phagocytosis, anti-inflammatory cytokine release and increase in production of the ECM. These are associated with tissue repair (82, 83). The ability of macrophages to rapidly shift based upon of stimuli represents a potential target for migraine, especially since they have functional CGRP receptors.

Macrophage function is intertwined with its cell surface receptors, cytokines that are released and morphology (81). In the past macrophages have been thought as “undifferentiated,” then respond to stimuli around them. Following activation, they were thought to shift to an inflammatory M1 phenotype or an anti-inflammatory M2 phenotype (82). The M1 inflammatory phenotype is typically characterized by anti-microbial and anti-tumor functions (Figure 2). An early study found that upon stimulation with interferon gamma, cultured human macrophages produced increased levels of hydrogen peroxide (84). This became known as the M1 phenotype. M1 macrophages produce other reactive oxygen species (ROS), release cytokines such as IL1-β to recruit other immune cells and change expression of genes to respond appropriately to the threat (85, 86). The release of ROS-related compounds can harm pathogens (86). When macrophages shift to the M1 phenotype, they change to a more circular appearance and may have reduced mobility (87).

The M2 phenotype of macrophage activation is generally thought of as anti-inflammatory. These macrophages function to ensure wound repair, engulf debris through phagocytosis, promote neovascularization and interact with the ECM (83, 88). M2 macrophages also express cytokines that are thought to fight inflammation. Compounds released by M2 macrophages include IL10and TGF-β (89). There have been significant advancements in understanding the complexity of activation state. Macrophages have multiple M2 sub phenotypes such as M2a, M2b, M2c and M2d (81). In fact, it is possible to manipulate the cell shape and cause more characteristics of the phenotype associated with the shape (90).

The adaptability of macrophages and their constant change in location, morphology and cell processes might be one way in which the dysregulated trigeminovascular space is caused. A preclinical study sought to examine macrophage activity through *in-vivo* two photon imaging (91). This was done in anesthetized animals under a two-photon scope. The macrophages and other CX3CR1-expressing cells produced green fluorescent protein, allowing visualization of the cells and their morphology. The team found that macrophages adopted an increased circularity following CSD in the mouse brain. They also saw a reduction in movement of dendritic cells. Changes in morphology is a potential but not absolute sign that the macrophages are being activated into one of the phenotypes (92). The macrophages were closely associated with transient receptor potential vanilloid receptor 1-positive nerve endings, suggesting close association with the trigeminal afferents (92, 93). This corresponds with *in vitro* work showing functional communication between cultured trigeminal neurons and macrophages (80). Similar association was also found in immunohistochemical analysis of rat dura maters. Macrophages appeared near dural vessels, and a portion were associated with afferent nerve endings (94). All this points to macrophages having possible crosstalk with trigeminal afferents, especially given the high portion of dural cells being macrophages (79).

In the context of migraine, macrophages can respond to several compounds released by trigeminal afferent endings, such as CGRP. Macrophages in culture have been shown to express the CGRP receptor and treatment of cells with CGRP causes an increase in cAMP (indicative of CGRP receptor activation) (95). If there is an initial CGRP release from trigeminal afferents, then that could potentially activate the dural macrophages. Reciprocal release of cytokines from the now-activated macrophage could sensitize the nearby afferent endings. This is known as neurogenic inflammation (27). This type of inflammation via endogenous compounds such as cytokines or CGRP is also referred to as sterile inflammation since there is a lack of an exogenous pathogenic trigger such as microbial membrane components (96).

The ultimate question is that even if CGRP binds to macrophages and increases cAMP levels, what is this doing to the macrophage itself regarding polarization? A recent pattern in studies observing macrophages is that CGRP appears to be preventing inflammation when cells are pretreated with a compound such as lipopolysaccharide (LPS) (97). In a study of mouse lung cells *in vitro*, lung macrophages were treated with LPS, and treatment with CGRP afterwards significantly reduced expression of inflammatory genes NLR family pyrin domain containing 3 and the pre-spliced IL1-β (97). Another study examined the role of CGRP-deficiency in bone marrow derived macrophages in the context of surgical implants (98). Cultured cells in the CGRP-knockout group had a far higher portion of CD86-expressing macrophages. This is generally considered a marker for M1 polarization (99). Supplementation in the knockout mice with CGRP reduced the M1 population and increased the M2 population, closer to those found in the wildtype controls. The results suggest that CGRP may be necessary for basal macrophage functioning, and a loss of CGRP results in a shift toward inflammation.

Another hint at CGRP's involvement in macrophages is with wound healing. In a recent study, CGRP's role in recovery was examined in corneal tissue. Cultured trigeminal ganglion neurons were co-cultured with macrophages in a rodent model with bacterial infection of the corneal nerves by *P. aeruginosa* (74). The research team found CGRP release from both macrophages and trigeminal ganglion cells when cultured separately following LPS administration. Together, they had a higher level of CGRP production following LPS than alone. CGRP prevented inflammation by promoting macrophages to express anti-inflammatory cytokines and markers. CGRP appears to be beneficial following infection of corneal afferents, and may help the local immune function, at least in the cornea. Another study found that CGRP levels were elevated in premolars from patients suffering from occlusion trauma in addition to orthodontic trauma (100). Other peptides were elevated, such as substance P and vascular endothelial growth factor. CGRP may be essential for tissue remodeling and wound healing acting as an activator or signal to macrophages in damaged tissue.

### Mast Cells

Mast cells are another major portion of the innate immune system, and the dura mater hosts these cells (67, 101). These cells originate from bone marrow precursors, like macrophages, but are not of monocytic lineage (102, 103). They are referred to as granulocytes due to their ability to release granules containing compounds such as histamine, tryptase, heparin, substance P and numerous cytokines (72). These chemicals are thought to mediate part of the neurogenic inflammation portion of migraine (60, 104). These compounds can act on subtypes of trigeminal afferents to sensitize them directly (105, 106).

In the context of migraine neurobiology, both CGRP and substance P are capable of directly acting on mast cells to induce degranulation (107–109). However, CGRP didn't activate human mast cells in the study by Kulka in 2008 while substance P did (108). Treatment of single mast cells with immunoglobulin E potentiated CGRP-induced degranulation, but not substance P-induced degranulation in culture (66). It is possible that while CGRP is indeed capable of inducing degranulation in mast cells, a priming event is needed. Since substance P is released along with CGRP from trigeminal afferents, it could be that substance P and CGRP are working synergistically to cause sustained degranulation. More studies are needed to assess the role of CGRP-induced degranulation. Components of the CGRP receptor have been colocalized to mast cells, but the degree of functional receptor presence is not well-known.

Even if CGRP's role in degranulation is less clear than other neuropeptides, it still can interact and induce neurogenic inflammation. Preclinical work in rodents found that treatment of animals with the mast cell degranulating agent 48/80 given via intraperitoneal injection at 2 mg/kg increased firing of trigeminal afferent endings present in the dura mater (67). The C fibers, which are unmyelinated afferents that release CGRP, had a higher increase in firing than the A delta afferent fibers. Increased firing of C fibers could release more neuropeptides and thus have a bidirectional relationship with the local dural mast cells. Besides CGRP or substance P, mast cells respond to numerous compounds such as allergens, cytokines, LPS, chemokines and others (110). The diversity of agonists for mast cells is important for the innate immune system, but in the case of migraine patients and immune disorders it may negatively affect the trigeminovascular space. A common irritant acrolein is capable of degranulating mast cells and is a known headache trigger (111, 112).

**Figure 3:**
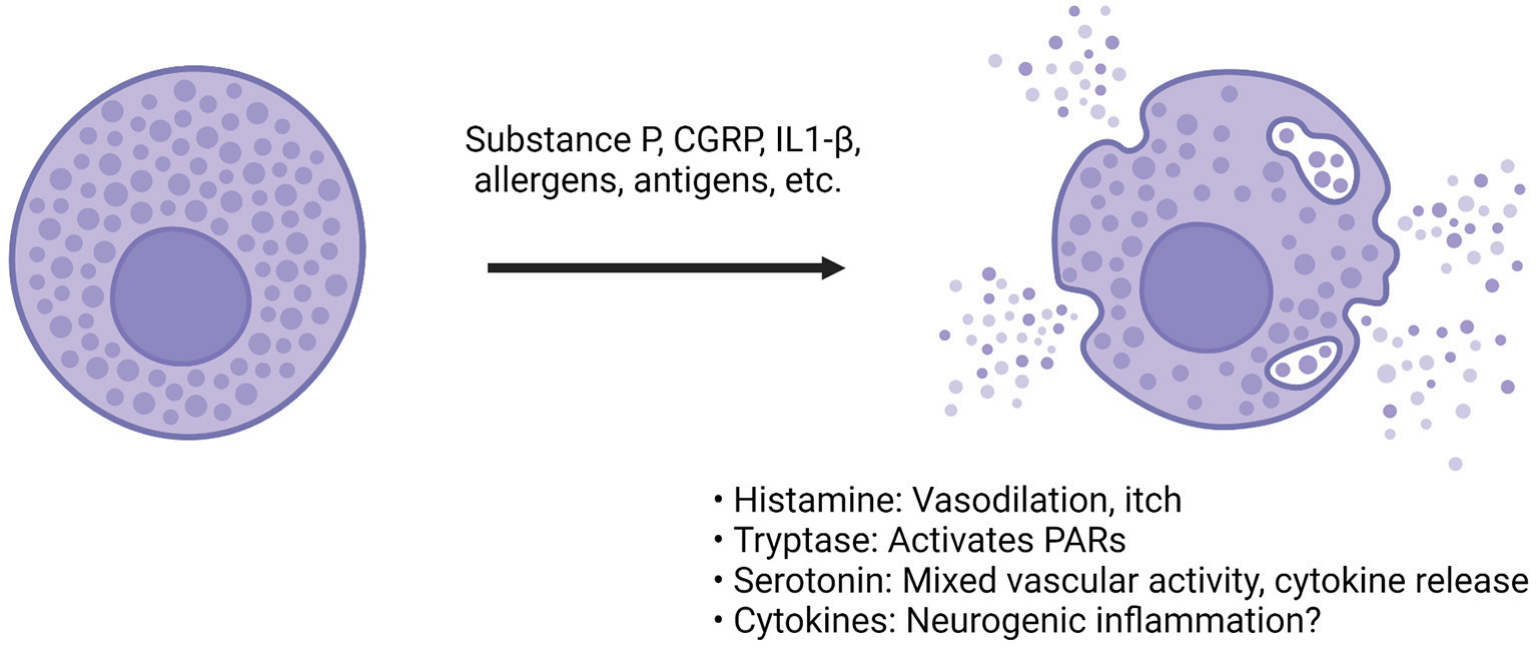
CGRP actions on dural mast cells. Compounds such as CGRP, substance P or inflammatory cytokines can bind to mast cells via a diverse array of receptors (66, 108). Following activation, the mast cell releases granules that contain compounds such as histamine, heparin, enzymes like tryptase, many different cytokines and serotonin (68, 72, 113). The effect of these compounds is diverse. Among the most prominent are histamine and serotonin. Histamine can cause vasodilation and immune activation. Serotonin has mixed vascular activity depending on the site of action. It has been found to dilate middle meningeal arteries (114). It can also modulate cytokine release from monocytes (115). Tryptase is a protease that can cleave other proteins, such as protease-activated receptor (PAR) 2. PAR-2 activation is thought to sensitize transient receptor potential channels present on sensory afferents, so downstream protease activity could possibly result in TG sensitization (116). The cytokines found in mast cells are incredibly diverse, and both pro- and anti-inflammatory cytokines have been found but degranulation is believed to drive further inflammation and sensitization of afferents (117).

Degranulation of mast cells releases a diverse number of chemicals and enzymes into the extracellular space (Figure 3). The most understood, histamine, is a known vasodilator (118). However, culture work on trigeminal afferents shows that treatment with histamine only resulted in a small increase in firing rate compared to compounds such as serotonin (113). Histamine was also found to promote an M2 phenotype like those seen in tumor associated macrophages, and prevention of histamine receptor H1 alleviates this shift in phenotype (119).

Other components released from mast cell degranulation include serotonin, heparin, tryptase, and numerous cytokines. Serotonin's effects on the dura and trigeminal neurons are complex, but can be summarized as increase in trigeminal firing, both vasoconstriction and dilation (depending on receptor subtype and concentration) and various immune actions, including secretion of specific cytokines (113, 115, 120, 121). For example, serotonin was found to dilate the feline middle meningeal artery, while triptans (5HT1_B/D_ agonists) caused vasoconstriction (20, 114). Tryptase is of interest because it is a serine-threonine protease capable of inducing changes to the extracellular environment and inducing nociception (122, 123). Researchers found that tryptase can induce mechanical allodynia is a mouse paw, and it does this through cleavage of proteinase-activated receptor (PAR) 2 (123). Antibodies against PAR-2 have shown beneficial effects in rodent models of pain and will be discussed later (124). Cytokines found in mast cells are conflicting, in part because of differences of measuring mRNA vs. functional protein (117). Numerous pro- and anti-inflammatory cytokines have been identified but more work needs to be done to parse out their relevance. Of the cytokines potentially released from mast cells, IL-1β and IL-6 have the potential to activate TG neurons or induce prolonged sensitization. In cultured rat TG cells, IL-1β increased prostaglandin 2 synthesis and subsequent CGRP release (125). In rodents, dural injection of IL-6 induced both facial and hind paw allodynia, demonstrating that local actions of cytokines in the dura can also apparently lead to central sensitization (126).

### Dendritic Cells

Dendritic cells are another type of monocyte lineage cell found throughout the body, including the dura mater (127). While they are in far lower proportion to macrophages, at least in rodents, they still have a substantial presence (79). These cells are involved in the antigen presentation process for B and T cells for adaptive immunity (128). They are a type of antigen presenting cell (APC). APCs take fragments of viruses and bacteria and return them to T cells. The T cells recognize fragments of the epitope and can differentiate accordingly (73). Dendritic cells treated with CGRP have a change in cell surface receptors and a decrease in proliferation, suggesting a functional CGRP receptor on these cells (75). Cultured dendritic cells have reduced cell migration following application of CGRP (129). In the skin, aggregation of dendritic cells in the lymph nodes was inhibited by CGRP, consistent with previous studies (130). A recent review summarizes CGRP's inhibitory effect on dendritic cells, reduced migration and antigen presentation (131).

Dendritic cells may be a potential target for headache and migraine treatment. A group in 2019 found that when dendritic cells were cultured from rats and exposed to interferon gamma, the exosome extract reduced spreading depression in hippocampal slices *in vitro* (132). The reduced electrical activity suggests that mild exposure of dendritic cells to specific cytokines may suppress neuron firing downstream, and they suggest that this could be one way to help patients with migraine.

### B Cells

B cells are another part of the adaptive immune system and can produce antibodies against specific antigens presented to them. Until recently, it was not known if the meninges had a substantial portion of B cells. In 2021, two papers reported a surprising abundance of B cells in the meninges, at least in rodents (133, 134).

CGRP's involvement with B cells is not well-studied, but CGRP was found to be expressed in these cells upon stimulation by nerve growth factor (NGF) (135). Levels were much lower in inactivated cells. B cells also appear to have CGRP receptors. B cells can respond to treatment with CGRP *in vitro*, and CGRP hinders development of B cell precursors. Blocking CGRP receptors with the peptide antagonist CGRP8-37 prevented this CGRP-driven inhibition (136).

### T Cells

Part of the adaptive immune system, T cells are responsible for circulating throughout the body and attacking antigens based upon antibody recognition and absorbing fragments of the pathogen. These cells are found in the dura. Based upon cell surface receptors, they can be divided into CD4 and CD8 cells (137). The CD4 T cells can be further subdivided into Th1 or Th2 (137, 138). Th1 is seen as pro-inflammatory, primarily through interferon gamma. Th2 is anti-inflammatory. The baseline level of dural T cells remains relatively low, while in bacterial infection there is a significant increase (139). Immunohistochemical analysis found that T cells were present around dural sinuses, with multiple phenotypes, such as Th1 or Th2 (140). Dural T cells increased as mice aged and had differentially expressed transcripts, such as interferon gamma. Multi-photon imaging confirmed the accumulation of T cells in the dura mater in transgenic mice (140). The researchers postulate that the accumulation of T cells at sinuses may represent an avenue for peripheral meningeal immunity through interactions with the CNS.

Dural T cells recognize centrally derived antigens. APCs in the dura such as dendritic cells and macrophages are capable to transport proteins in the CSF to T cells (140). T cells appear to be able to respond to CGRP, suggesting presence of a functional receptor (141). CGRP-stimulated dendritic cells also can shift T cells to the Th2 phenotype (130). So CGRP may have multiple avenues to influence T cell functioning.

## Discussion

**Figure 4:**
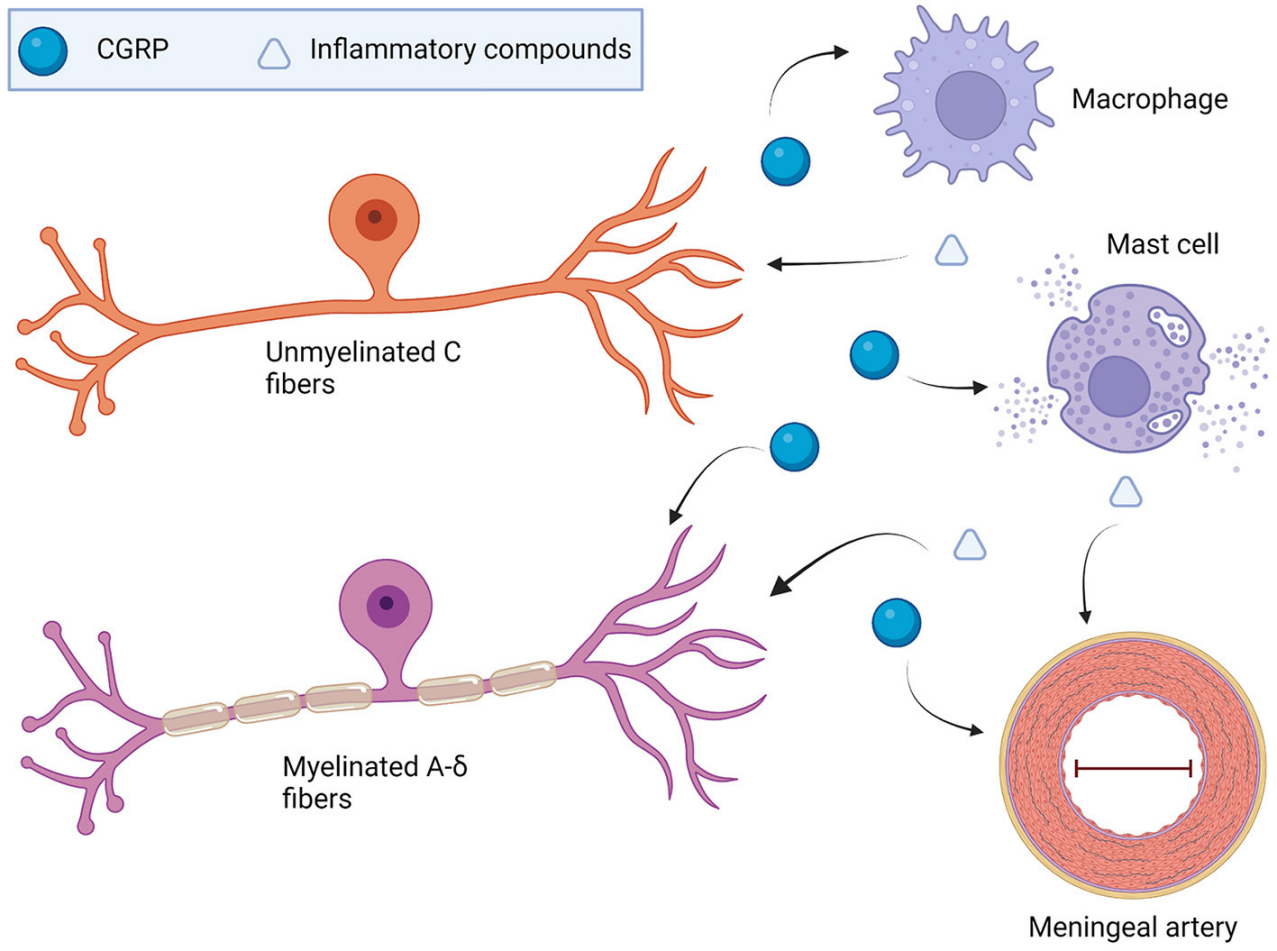
Model of CGRP involvement with the dural immune system in migraine. TG-produced CGRP may drive dural mast cell degranulation that could sensitize nearby TG afferent endings. It could also result in vasodilation and activation of macrophages and other resident immune cells. Beyond the direct activation of TG afferents by inflammatory compounds, vasodilation via histamine or neuropeptides might activate mechanoreceptors present on TGs. Mechanoreceptor activation such as Piezo2 present on sensory neurons could be one way sensitization of TG neurons occurs. Macrophages could change the extracellular makeup on the dura following polarization from neuropeptides or cytokines. *in vitro* work shows that CGRP shifts either LPS-treated macrophages or tissue damage macrophages toward an M2 tissue-remodeling phenotype (65, 97). Changes to the interaction of the TG endings and the dura superstructure could potentially cause dysregulated firing via mechanoreceptors.

The immune system within the dura mater is extensive and dysregulation is likely to contribute to migraine pathophysiology. CGRP is a multifaceted neuropeptide that can modulate these dural immune cells. CGRP can induce neurogenic inflammation through mast cell degranulation and subsequent inflammatory chemical release. CGRP actions are likely to extend beyond mast cells, with recent articles hinting at CGRP being vital for shifting macrophages toward an anti-inflammatory phenotype following stimulation with either LPS or injury. CGRP also has a generally inhibitory effect on dendritic cells through reductions in migration and antigen presentation. Hence, CGRP cannot be pigeonholed as either a pro- or anti-inflammatory peptide since it is inflammatory via mast cells and anti-inflammatory through macrophages and dendritic cells. Figure 4 presents a potential model for the role of CGRP and other neuropeptides on dural mast cells and macrophages. These are the two most well-studied immune cells in the context of CGRP. However, a caveat of this model is that most studies on CGRP and immune cells have been with either cell lines or bone marrow-derived immune cells, not immune cells in the meninges. Whether meningeal immune cells will respond in the same manner to CGRP remains to be determined.

CGRP-targeting medication is effective in 50% of chronic and episodic migraine patients, but some recent studies have brought on concerns about potential immune effects. A series of 8 case studies from Australia and Ireland found potential immune complications from the CGRP antibodies erenumab or galcanezumab (142). Patients had complications such as a man who suffered from rheumatoid arthritis. After taking the antibody treatment, he had hepatitis. Cessation of erenumab helped him recover, along with steroid treatment. Another patient had psoriasis upon treatment with CGRP antibody. Another case study examined a 51-year-old woman who following erenumab treatment had skin infiltration of lymphocytes and thrombosis. While these are only case studies, this provides hints at CGRP's multifaceted, and nuanced role in human immune physiology.

Another aspect to consider is the response of the immune system to CGRP's blockage over time. We don't yet know the impact on the immune system if CGRP is blocked for years, but within a year timeframe of CGRP antibody usage there are apparently no major complications in most patients (143). A further complication of CGRP's role on dural immune cells is the ability of the immune cells to both respond to CGRP, and to also release it upon stimulation by certain chemicals. Studies have found that macrophages and B cells can produce CGRP (135, 144). So neural-produced CGRP might affect downstream CGRP production in these cell types, resulting in a potential feedback loop between neurons and immune cells. It is unknown if immune cell produced-CGRP has a physiological role but it does represent another layer of complexity of the system. In addition, other cell types may produce CGRP besides immune cells (145). Thus, both neural and locally expressed CGRP in the meninges is well-poised to modulate the activities many types of immune cells and possibly contribute to migraine pathogenesis.

## Conclusion

The complexity of the neurobiology of migraine perplexed researchers for years, yielding the vascular vs. neuronal debate. While the neuronal side is favored as of late, it is only a part of the system. The neuropeptide CGRP is clearly involved in the genesis and continuation of migraine headache for many patients. Therapeutics targeting CGRP or the receptor are effective, but only in ~50% of episodic or chronic migraine patients, which illustrates the need for a better understanding of how CGRP contributes to migraine pathogenesis. CGRP has primarily been viewed as a pro-inflammatory molecule in most migraine literature, but it also has anti-inflammatory and tissue repairing functions through macrophages, dendritic cells, B and T cells. CGRP is cardioprotective and helps healing following injury. There is evidence of immune dysfunction in migraine. More work should be done examining CGRP's role in not just migraine neurobiology, but also its function in the immune system. Targeting CGRP's role in migraine immune dysfunction could both augment CGRP therapeutics and possibly help patients who are non-responders to drugs that target only CGRP or its receptor.

## Author Contributions

LB wrote the article and made the figures, with thorough editing from AR and guidance on the layout of the figures. All authors contributed to the article and approved the submitted version.

## Funding

AR was supported by NIH R01 NS075599.

## Conflict of Interest

AR is a consultant for Lundbeck, Eli Lilly, AbbVie, and Schedule One Therapeutics. The remaining author declares that the research was conducted in the absence of any commercial or financial relationships that could be construed as a potential conflict of interest.

## Publisher's Note

All claims expressed in this article are solely those of the authors and do not necessarily represent those of their affiliated organizations, or those of the publisher, the editors and the reviewers. Any product that may be evaluated in this article, or claim that may be made by its manufacturer, is not guaranteed or endorsed by the publisher.

## Abbreviations

IHS: International Headache Society
CSD: Cortical spreading depression
CGRP: Calcitonin gene-related peptide
TG: Trigeminal ganglion
CNS: Central nervous system
IL1-β: Interleukin 1-beta
IL6: Interleukin-6
IL10: Interleukin 10
TNF-α: Tumor necrosis factor α
CSF: Cerebrospinal fluid
TGFβ-1: Transforming growth factor beta-1
IBS: Irritable bowel syndrome
GI: Gastrointestinal
SNP: Single nucleotide polymorphism
IL4: Interleukin 4
ECM: Extracellular matrix
ROS: Reactive oxygen species
LPS: Lipopolysaccharide
PAR: Proteinase-activated receptor
APC: Antigen presenting cell
NGF: Nerve growth factor.
